# A Comprehensive Compilation of Graphene/Fullerene Polymer Nanocomposites for Electrochemical Energy Storage

**DOI:** 10.3390/polym15030701

**Published:** 2023-01-30

**Authors:** Judy Gopal, Manikandan Muthu, Iyyakkannu Sivanesan

**Affiliations:** 1 Department of Research and Innovation, Saveetha School of Engineering, Saveetha Institute of Medical and Technical Sciences (SIMATS), Chennai 602105, India; 2 Department of Bioresources and Food Science, Institute of Natural Science and Agriculture, Konkuk University, 1 Hwayang-dong, Gwangjin-gu, Seoul 05029, Republic of Korea

**Keywords:** energy storage, electrochemical, graphene, fullerenes, carbon

## Abstract

Electricity consumption is an integral part of life on earth. Energy generation has become a critical topic, addressing the need to fuel the energy demands of consumers. Energy storage is an offshoot of the mainstream process, which is now becoming a prime topic of research and development. Electrochemical energy storage is an attractive option, serving its purpose through fuel cells, batteries and supercapacitors manipulating the properties of various materials, nanomaterials and polymer substrates. The following review presents a comprehensive report on the use of carbon-based polymer nanocomposites, specifically graphene and fullerene-based polymer nanocomposites, towards electrochemical energy storage. The achievements in these areas, and the types of polymer nanocomposites used are listed. The areas that lack of clarity and have a dearth of information are highlighted. Directions for future research are presented and recommendations for fully utilizing the benefits of the graphene/fullerene polymer nanocomposite system are proposed.

## 1. Introduction

**Figure 1:**
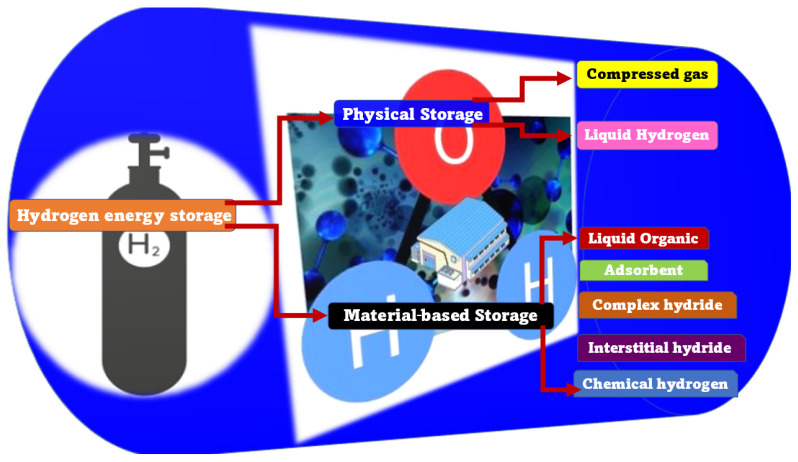
List of various storage methods for hydrogen storage.

Human society depends largely on electrical energy for its routine day-to-day activities. A vast quantity of the available energy is procured from chemical energy stored in fossil fuels. Coal and natural gas are used to generate electricity by combustion (thermal power). In addition, heat energy generated during nuclear fission of uranium is also used for generating electricity (nuclear power). These are non-renewable sources. Electrical energy is also tapped from six major renewable resources: hydro, wind, photovoltaic, concentrating solar, geothermal and biomass power. As humanity is close to exhausting the stored fossil fuels that are non-eco-friendly, technological improvements to tap energy, especially from alternative renewable resources such as hydroelectric, geothermal, solar and wind, have led to a rapid increase of power generation across the globe. In the United States, a recent report on renewable energy shows that it shares 11% and 17% of the total energy demand and the total electricity generation, respectively, according to the U.S. Energy Information Administration, 2017 [1]. Further, in China, out of its total power installations, 38.4% belong to power generation from renewable energy sources, which contributed up to 26.7% in 2018 [2]. In India, 21% of the total installed power capacities belong to renewable energy sources, such as small hydro, wind, biomass, WTE and solar power [3]. It was predicted that the total global contribution of renewable energy to the primary energy supply (14% in 2015) and to the power sector (25% in 2015) will have been raised to 63 and 85%, respectively, by 2050 [4]. Hydrogen energy is an attractive alternative, which uses hydrogen and/or hydrogen-containing compounds to generate energy. Its benefits include high energy-efficiency, environmental and social benefits and economic competitiveness. The world is currently experimenting with implementing hydrogen energy in various sectors, and hydrogen storage is, once again, the major challenge. Figure 1 lists the various storage methods that are available for hydrogen storage.

Although these renewable energy sources contribute to a considerable amount of global energy production, storage is an important aspect. Therefore, much emphasis has been given to the development of electrochemical energy storage devices, such as fuel cells, batteries and super capacitors. Batteries are electrochemical cells which have the capacity to deliver electrical current; the voltage of the batteries purely depends on the size and number of electrochemical cells connected in a series [5]. Batteries are used in numerous applications that require storage [5]. Batteries with conventional metal and inorganic materials such as lead-acid batteries, lithium-ion (Li-ion) batteries, sodium-sulfur batteries (NAS), flow batteries and Zn-air batteries, have been found to be suitable for commercial applications [6]. Lithium-ion batteries and nickel-cadmium batteries are renowned for their best storage capacities and widespread usage [7,8,9]. Though batteries are capable of better energy storage, their thermal instability and inadequate cycle life are major limitations. In addition, toxic leakages from batteries are hazards to the environment as well as to the users and, therefore, batteries with environmentally friendly materials having high energy-storage capacity, such as polymers (which predominantly are eco-friendly), have been sought as an eco-friendly alternative. 

Supercapacitors, otherwise called electrochemical double-layer capacitors (EDLC) are yet another class of electrochemical devices that store electrochemical energy on porous electrode surfaces under the influence of electrolytes [10]. EDLCs are otherwise known as super capacitors or ultra-capacitors. EDLCs are electrochemical capacitors that employ conducting polymers as electrodes. An EDLC enables large power effects up to 10 kW/kg with 10 Wh/kg storage capacity short storage time of 30–60 s. A 1-m^3^. EDLC is expected to yield 1–5 MW power pulse and weighs 100–500 kg [11]. The price is around 200–600 €/kWh and 50–150 €/Wh, but within a decade, a reduced price of 10–15 €/Wh is predicted. The most important limitation of EDLCs is their high cost [12]. Supercapacitors are far superior to batteries when it comes to their charge/discharge cycles; however, their low energy density limits their practical applications [13]. Therefore, their electrochemical storing capacities are being further developed using carbon-based electrodes. Various carbon-based nanomaterials such as carbon nano tubes [14,15,16,17,18], graphene and fullerenes have been used as supercapacitor materials for improved storage of electrical energy [11,12,19,20,21].

In the present review, we survey the progress achieved through the use of carbon polymer nanocomposites for electrochemical energy storage. The use of graphene polymer nanocomposites and fullerene polymer nanocomposites for electrochemical energy storage are reviewed, and the various combinations of their respective nanocomposites and the progress made in terms of energy storage are presented. The challenges and the lapses in the existing knowledge are addressed and recommendations proposed.

## 2. Carbon Polymer Nanocomposites Used for Energy Storage

Recently, the topic of energy has inspired a great deal of interest and curiosity, as the world’s primary energy sources are being increasingly depleted due to rising home and industrial energy consumption. This has increased the demand for efficient, environmentally friendly and economically viable energy sources. Devices for energy conversion and storage that are affordable and efficient are in high demand due to the world’s expanding population. Materials made of graphene or polymers have benefits including high specific active surface areas, great electron transport capability and good capacitance. They are frequently used as lithium-ion batteries, electrode materials for fuel cells, and other devices. Lithium-ion batteries, sodium-ion batteries, potassium-ion batteries, lithium-air batteries, supercapacitors and other devices are examples of new energy storage technologies. The electrochemical cells of batteries and supercapacitors store energy chemically. In order to ensure appropriate and reliable devices that can store a sufficient amount of energy for transportation, electronic devices, electric-powered carriers and various other purposes, energy storage methods require uniquely authentic storage approaches. Supercapacitors, a variety of batteries and fuel cells are all examples of electrochemical energy storage devices [22]. Thermal, compressed air and flywheel energy storage are other forms of energy storage [23,24,25].

**Figure 2:**
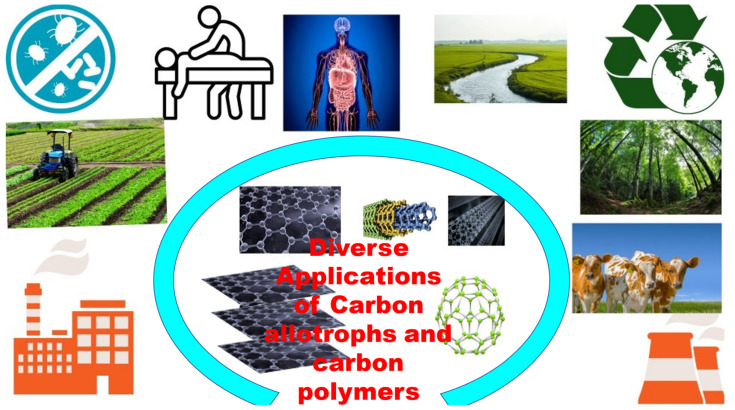
The varied applications where carbon allotrophs and carbon polymers are exploited.

Carbon-based polymer nanocomposites (CPNCs) are used in a variety of industries, including aerospace, automobiles, packaging, energy storage and energy accumulation [26,27]. Figure 2 presents the multifarious carbon nanomaterial applications. These nanostructures possess unique properties, such as ease of processing, adaptability to configurations, lightweightness and flexibility. These materials have found their use in the making of the future of renewable energy, namely, fuel cells and supercapacitors for energy storage [28,29]. Novel nanocomposite materials for automotive and electric energy storage applications have been discovered for inexpensive devices with high energy and power densities. Insulating polymers loaded with high-aspect-ratio conductive nanofillers, such as carbon nanotubes (CNT) and graphene nanoplatelets (GNP) [17,18,30,31], have been demonstrated as potential dielectric materials [32,33]. Space charge polarization significantly improved when nanocomposites were used in an insulating pattern [34,35,36,37].

Energy storage innovations from renewable energy sources are being sought to offset the current energy-related issues that arise from the use of traditional energy sources. It is urgently necessary to develop environmentally acceptable energy solutions; therefore, the electrochemical storage of energy involving electrochemical capacitors, batteries and FCs are gaining increasing popularity [38]. Nanocomposite materials with superior qualities are now available, among which, carbon polymer nanocomposites offer a wide range of opportunities [39].

CNTs are the most representative nanocarbons, which are known for their outstanding electrical properties, strong mechanical strength, high chemical stability, high aspect ratios and higher activated surface areas. The potential of CNTs as electrodes in Li-ion batteries has been proposed and tested by many research groups as early as the 1990s [40,41,42,43,44,45]. Even though carbon materials possess lower ED because of their adsorption response, the creation of EDLCs, carbon materials such as activated carbon (AC), carbon nanotubes (CNTs) and graphene nanosheets (GN) have been extensively studied for their suitability for supercapacitor applications. 

**Figure 3:**
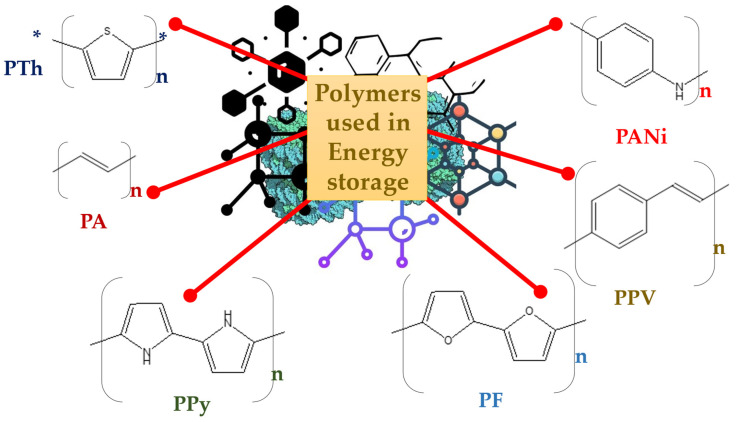
Structures of polymers that are commonly associates with carbon allotropes for energy applications.

In contrast to batteries, which have yields that are limited by their sustained chemical power, FCs are electrochemical processes that constantly produce electricity while the fuel (for example, H_2_) appears to be an oxidant. Fuel cells are generally classified as phosphoric acid fuel cells (PAFC), polymer electrolyte membrane fuel cells (PEMFC), alkaline fuel cells (AFC), molten carbonate fuel cells (MCFC) and solid-oxide fuel cells (SOFC), based on the type of electrolytes used. Fuel cells that use polymer electrolyte membranes (PEMs), such as direct methanol fuel cells (DMFCs) and PEMFCs, for example, are the norm for serving applicants with low-temperature performance. Typically, FCs are grouped based on their construction, working conditions (such as temperature), and the FCs’ polymer electrolytes’ properties [46]. A recent review by Chen et al., 2022, summarized the recent developments in carbon-based nanocomposites for fuel cell applications [47]. In their review, they elaborately explained the usage of several forms of carbon nanomaterials, such as carbon aerogels, carbon nanofibers, graphene, carbon nanotubes and fullerenes, in the development of hydrogen fuel cells. They also described the various principles, reaction mechanisms and cyclic stability of the fuel cells, as well as new strategies and future challenges related to the development of viable fuel cells. Among the traditional ED techniques used for electrochemical energy storage, lithium-ion batteries are among the most significant. Due to Li-ion batteries’ effectiveness and configuration flexibility, they are widely used for electricity storage. Due to their special qualities, which include strong electrical performance and good coulombic efficiency, carbon polymer nanocomposites promote organic-inorganic composites for use in Li-ion batteries. This encourages the periodic usage of batteries with mild deterioration in their construction. Polyaniline (PANI), Poly(3,4-ethylenedioxythiophene) (PEDOT) and Polypyrrole (PPy) would ideally be the polymers most integrated with carbon nanomaterials. Figure 3 gives the structures of the polymers that are predominantly associated with carbon allotropes for energy applications. When used as electrodes, carbon polymer nanocomposites (CPNCs) demonstrate a number of advantages, including superior processability, reduced cost, tolerable molecular change and lightweightness. Although CPNCs can be used as anodic or cathodic materials, Li-ion batteries frequently use them as cathodes. Different CPNCs exhibit remarkably different EDs, for instance, PPy-based probes have EDs of approximately 10–50 Wh kg^−1^ and PDs of about 5–25 kW kg^−1^, PANI-based probes produce EDs of about 50–200 Wh kg^−1^, and PTh-based probes give EDs of 20–100 Wh kg^−1^, and PDs of 5–50 kW kg^−1^. The capability and rate recitals of the Li-ion battery have recently been established using carbon-based composite probes that use CNTs [48]. 

Sarang et al. [49] investigated the n-type redox reaction with poly-fluorene-alt-naphthalene diimide (PFNDI). A microwave-assisted solvothermal process was used at liquid-phase exfoliated GN (LEGr) to create the GN@SnS_2_ heterojunction nano molecule [50]. After over 200 additional cycles with a 300 mA/g current density, the storage capacity still was maintained at 664 mAh/g. Pseudocapacitors and EDLCs are the two types of SCs. In EDLCs, energy is conserved electrostatically on the probe and conducting solution edge into the double layer, whilst the pseudocapacitor charge storage occurs through rapid redox reactions at the electrode exterior. Carbon-based materials, MOx/hydroxides, and CPNCs are the three main categories of conductor materials for SCs in this context [51]. CPNCs have excellent PD and a long life cycle.

The PPy/CNT combination has been used as a reliable pseudocapacitive cathode for non-aqueous LIC applications. The inclusion of CNT greatly enhances electrical performance, while the PPy exhibits strong pseudocapacitance due to the doping/undoping effect. In its current state, the composite outperforms porous carbon negatives found in current LICs in terms of capacities and stability (98.7 mAh g^−1^ on 0.1 ag^−1^, plus holds 89.7% after runs on 3 ag^−1^ for 1000 cycles). Additionally, when connected to the Fe_3_O_4_@C positive electrode, the as-developed LICs exhibit a higher ED of roughly 101.0 Wh kg^−1^, on 2709 Wh kg^−1^, while still maintaining 70 Wh kg^−1^ through an enhanced PD of 17,186 W kg^−1^ [52].

Due to their good specific surface area and exceptional electrical and mechanical properties, carbon nanocomposites—in particular, CNTs plus GN—have recently been thoroughly investigated as active electrodes in SCs. According to recent research, high-performance SCs can be outfitted with electrodes based on vertically aligned CNTs and GN sheets constructed as 3D pillared GN-CNT systems. High electrical conductivities and high doping-dedoping rates are provided by electroactive polymers (polyaniline, polypyrrole, polythiophene and its derivatives) during charge–discharge operations. [12,53,54]. At operating voltages of about 3 V, these polymers exhibit high gravimetric and volumetric pseudocapacitance in a variety of nonaqueous electrolytes. However, these electroactive polymers normally exhibit poor mechanical stability. The electrode swells and contracts resulting in degradation of the cell, which impairs the electrochemical performance. Low-cost electroactive polymers typically have a limited life cycle when utilized as bulk materials for electrodes [37]. Improved mechanical qualities are demonstrated by electrodes composed of carbon polymer nanocomposite materials.

The effective synthesis of carbon polymer nanocomposites is an intriguing development for the fabrication of a new generation of supercapacitors. An electrodeposited polymer called polyaniline (PANI), a promising polymer for use as an electroactive material, has been supported by the previously stated hierarchically porous carbon monolith (HPCM). HPCM (surface area: 277 m^2^ g^−1^, pore volume: 0.47 cm^3^ g^−1^) was employed as a high surface support for conducting polymers, as well as a current collector. In a three-electrode cell experiment in 1 M H_2_SO_4_, a specific capacitance of about 2200 F g^−1^ (i.e., per gramme of PANI) was obtained at a current density of 0.67 A g^1^ in the potential range of 0–0.7 V with a power density of 0.5 kW kg^−1^ and an energy density of 300 Wh kg^−1^. The specific capacitance was still as high as 1270 F g^−1^, even at a very high current density of 66.7 A g^−1^. These numbers are all surprisingly high [55]. PANI electrodeposited onto a surface has a substantially lower specific energy density than PANI placed on HPCM. PANI electrodeposited onto a nonporous carbon monolith displays a considerably lower specific energy density than PANI placed on HPCM (abbreviated as NPCM). PANI and HPCM work together synergistically to produce the improved characteristics [56]. Additionally, pellet electrodes made by simply pressing nanocomposite materials have been claimed to be useful for supercapacitors, particularly when an asymmetric configuration is achieved [55] For instance, the specific capacitance of an asymmetric capacitor with polypyrrole/CNTs as the negative electrode and PANI/CNTs composite materials as the positive electrode, can reach a value of up to 320 F g^−1^. Because they combine two relatively inexpensive materials to acquire substantial pseudocapacitance, it appears that composites of nanocarbons with conducting polymers could be more appealing. It is not possible to directly compare the measured value of specific capacitance to that of a two-electrode cell. In a three-electrode cell, for instance, high values up to 1100 F g^−1^ have been recorded for a PANI/CNTs composite electrode. A substantially lower specific capacitance value of 360 F g^−1^ was measured using a two-electrode cell [55]. In the following sections, we will review the specific milestones achieved through graphene and fullerene carbon allotrophs. 

## 3. Graphene-Based Nanocomposites for Electrochemical Energy Storage

Recently, composites of polymers and nanofillers, such as graphene-based materials, have been welcomed as probes for boosting the activity of SCs by using the high synergistic impact. Due to their unique physical, morphological and structural characteristics, graphene-based systems are crucial components for energy applications [57]. Polymer/graphene nanocomposites have attracted great interest as cathode materials since polymers are sustainable, environmentally benign (“green” cathodes), have inherently faster kinetics and an electrochemically stable backbone, and can be paired with electrochemically active functional groups [58,59,60]. In this section, we give a brief overview of the various graphene-based nanocomposites that have been reported for electrochemical energy storage. These were made possible thanks to innovative nanotechnology that allowed for the creation of energy storage equipment using carbon-based nanomaterials like graphene, carbon nanosheets, AC, CAGs, MOx, carbon polymers and polymer amalgams. SC applications make use of PANI nanocomposites and carbon-based electroactive materials. By using CO_2_, GO (graphene oxide)/PANI-PANI nanoparticles with a high Cs (425 F g^−1^) was achieved. The interaction between GO (having a high specific surface area) and the nanosized PANI nanocomposites gives these materials their distinctive electrochemical capacitance and cycle durability [61].

Researchers have focused on poly(anthraquinonyl sulfide) and polyimide composites with graphene using in situ polymerization [62] A unique rGO/PANI/rGO double-decker composition nanohybrid paper was created by Xiao et al. [63] and its potential as an SSC probe was examined. The self-supporting GN paper was primarily created using the print method and dazzling delamination process. It displayed superior mechanical properties, good electrical performance (340 S cm^2^) and low weight. As a result, a sandwich-structured GN/PANI/GN paper was created. It is interesting to note that the probe’s energy storage capacity, rate execution and cycling durability were greatly enhanced. The as-achieved SSC, therefore, displayed an exceptional capacitance of around 120 mF cm^2^, which was confirmed at 62% after an increase of current density between 0.1 and 10 mA cm^2^, including an ED of about 5.4 mW cm^3^.

In another study, using NaOH as a co-precipitate and GO proton-rich component, a unique single-step approach was used to create a paired composite of GN integrating iron oxide (rGO/MeFe_2_O_4_). The rGO/MnFe_2_O_4_ compound probe, with a sweep rate of around 5 mV/s, showed a gravimetric capacitance of about 147 F g^−1^, including an oxidative capacitance of about 232 mFcm^2^. The ternary GN/metal-doped iron oxide/PPy (rGO/MnFe_2_O_4_/Ppy) compound probe displayed significantly higher gravimetric and oxidative capacitances of around 232 F g^−1^ and 395 mFcm^2^, respectively, demonstrating the combined effect of PPy [38]. By using 1 M NaCl media and integrating GN/PPy composite material, Biswas et al. [64] demonstrated a gravimetric capacitance of about 165 F g^−1^. According to Parl et al. [65], graphite/PPy compound was applied on SC electrodes with a gravimetric capacitance of 400 F g^−1^ [66] The gravimetric capacitance of the synthesized ternary PPy/GO/ZnO SC electrodes, which are arranged with two probes, was 94.6 F/g/Ag/g. Additionally, according to Lim et al. [67], a ternary PPy/GN/nano MnOx complex’s gravimetric capacitance was 320.6 F g^−1^ on 1 mV s^−1^, which was significantly higher than the gravimetric capacitances of straight PPy and PPy/GN, which were 255.1 F g^−1^ and 118.4 F g^−1^. Xiong and colleagues [68] calculated the gravimetric capacitance of ternary cobalt ferrite/GN/PANI nanomaterials, which resulted in a gravimetric capacitance of approximately 1133.3 F g^−1^ on the sweep rate at 1 mV s^−1^.

Gao et al. [69] examined the viability of using hybrid nanocomposites of graphene quantum dots (GQD) and dyes based on phenoxazine as an effective sensitizer for dye-sensitized solar cells (DSSC). According to Akhina et al. [70], plasticized poly (vinyl chloride) combined with rGO were employed to create flexible composites with high dielectric permeability and low dielectric loss. After the addition of RGO, the dielectric constant increased by 57% (vinyl chloride). This improvement could be due to the increased number of polymer and filler contacts. The vacuum filtration of GO and PANI dispersions created supercapacitor devices based on graphene/PANI composite sheets, which display significant electrochemical capacitance. Suneetha et al. [71] used an optimal quantity of each component to create a nanocomposite of Zinc-doped Iron oxide, Graphene Oxide and Chitosan. Cyclical voltammetry was used to analyze the composite’s electrochemical properties, while impedance tests were used to analyze its capacitive behavior (EIS). These electrochemical studies showed that the composite had high adhesion to the electrode surface at pH 1, and they also showed greater electrochemical stability with clearly defined redox peaks. The nanocomposite modified electrode displayed good capacitance with a phase angle of 87°, demonstrating its excellent suitability for supercapacitor applications. Chabi et al. [72] created 3D graphene foam (GF) with PPy functionalization that displayed exceptional electrochemical performance. The resulting 3DPPY GF electrode was employed directly as a working electrode without binder or carbon additions because it is free-standing. Due to the special characteristics of the PPY-GF composites, such as their highly conductible p-doped PPY and hierarchically flexible 3D network, the electrodes made with these materials have enhanced pseudo-capacitive capabilities. For use in supercapacitor applications, Azizi et al. [73] created a novel reduced graphene oxide/poly (1,5-dihydroxynaphthalene)/TiO_2_ (RGO/PDHN/TiO_2_) ternary nanocomposite conducting polymer. The RGO/PDHN/TiO_2_ nanocomposite polymer film displays a high specific capacitance of 556 F g^−1^, which is much higher than the values obtained using RGO/PDHN (432 F g^−1^) and PDHN (223 F g^−1^) at a current density of 2.4 A g^−1^. The substantial specific capacitance of RGO/PDHN/TiO_2_ is the result of the simultaneous use of the electrical double layer capacitance (EDLC) of RGO with the pseudocapacitive behavior of PDHN and TiO_2_. After 1700 cycles, the RGO/PDHN/TiO_2_ nanocomposite preserves around 74% of the initial capacitance values and exhibits longer self-stability than other polymers. 

Polymer electrolyte membranes (PEM) are a crucial component of energy conversion and storage devices such as fuel cells, electrolyzers and batteries. The development of GO membranes, their interaction with the polymer matrix and their electrochemical characteristics have made progress. By using GO, Si nanoparticles, polymer monomers (acrylic amide) and networking agents (*N*,*N*′-methylene bisacrylamide) as the raw materials, Pan et al. [74] prepared a 3D framework Si@N-doped C/reduced graphene oxide (Si@NC/rGO) composite. As an anode electrode material for LIBs, the Si@NC/rGO composite exhibits outstanding rate performance, strong cycle stability and a sizable reversible specific capacity. After 200 cycles, the specific capacity remains at 867.4 and 479.1 mAh g^−1^ at 0.1 and 2 A g^−1^, respectively. Compared to commercial graphite anode materials, the capacity is three to four times higher. The C structure, the high electrical conductivity of graphene and N doping work together to provide the exceptional electrical properties. The composite of Si@NC and rGO exhibits expansion potential as anode electrode materials for LIBs. For the production of rGO/polyaniline (PANI)/Pt-Pd composite, Arukula et al. [75] described a wet reflux technique. This composite was used as a potential anode catalyst with increased methanol oxidation capability for direct methanol fuel cells (DMFCs). 

Reduced graphene oxide (rGO)/poly(3,4-ethylenedioxythiophene): polystyrene sulfonate (PEDOT: PSS) nanocomposites exhibited higher anodic current density of 48 mA cm^2^, as well as improved cyclic stability of 93% at 800th cycles. The change in shape of the nanocomposite at the fluence of 3.3 1016 ions cm^2^ and the appearance of graphite-like clusters were responsible for improved electrocatalytic activity. A non-precious anode catalyst material PEDOT:PSS/MnO_2_/rGO ternary nanocomposite was created by Baruha et al. [76] via a hydrothermal method and in situ oxidative polymerization. The heterogeneous rate constant (ks) and anodic and cathodic electron transfer coefficients of the ternary nanocomposite-coated electrode were determined to be 0.51, 0.45 and 0.055 s^−1^, respectively. The excellent conductivity of rGO nanosheets and the porous nanostructure of PEDOT: PSS coated MnO_2_ nanorods may work in concert to increase the electrocatalytic activity of the ternary nanocomposite toward the oxidation of methanol, as measured by its higher oxidation current density (56.38 mA/cm^2^) and lower onset potential (0.32 V). The PEDT:PSS/MnO_2_/rGO ternary nanocomposite may be a suitable replacement for the platinum-based anode catalyst in direct methanol fuel cells due to its long-term stability retaining of current density 50 mA/cm^2^ up to 1 h and greater cyclic stability (current retention factor 83%) up to 700th cycles.

To further enhance the performance of graphene-based electrodes for supercapacitors, hybrid structures of graphene and electrically conducting polymers like polyaniline (PANi), polypyrrole (PPy), poly(thiophene) (PTh), poly(hexylthiophene) (PHTh) and poly(3,4-ethylenedioxythiophene) (PEDOT) are frequently used. Potential supercapacitor electrodes made of graphene-PANi nanocomposites have been studied [77,78,79] and exhibit significantly increased electrochemical performance. Graphene-PANi nanocomposites have good rate capability of 581.6 F g^−1^ at 5 A g^−1^ and high specific capacitance of 863.6 F g^−1^ at 0.2 A g^−1^ [79]. Flexible graphene-PANi nanocomposites with a specific capacitance of 1126 F g^−1^ and capacitance retention of 84% after 1000 cycles could be produced via an in situ polymerization-reduction/dedoping-redoping method [80]. By mixing 1D PANi nanowires and 2D GO nanosheets, Xu et al. presented a simple technique to create hierarchical nanocomposites [81] The nano-composite has a remarkable cycle life and a specific capacitance of more than 550 F g^−1^ at 0.2 A g^−1^ current density [81] Other hybrid materials have also been considered as potential materials for supercapacitors, including flexible graphene-PANi nanofibers, graphene-PANi flakes, PANi-embedded holey graphene nanoribbons, hierarchical graphene@PANi@graphene sandwich containing hollow structures [82], 3D porous graphene/PANi [83], freestanding hierarchical CNF/GO/PANi [84]. Another conductive polymer utilized in supercapacitors along with graphene is PPy. At a current density of 0.5 A g^−1^ in 1 m H_2_SO_4_, polymerized PPy with graphene displayed a high specific capacitance of 482 F g^−1^. At a discharge rate of 0.5 A g^−1^, sulfonated graphene (SG) and PPy composite films with 40 wt% of SG had a specific capacitance of 285 F g^−1^. With 95% capacity retention after 1000 cycles at a scan rate of 100 mV s^−1^, hierarchical graphene-PPy nanosheet nanocomposites provided a capacitance of 318.6 F g^−1^ at a scan rate of 2 mV s^−1^. As an electrode for supercapacitors, composite films made of PPy and GO were electrochemically created. The GO/PPy composite had a high specific capacitance of 424 F g^−1^ in 1 m H_2_SO_4_-based electrolytes at a current density of 1 A g^−1^ [85]. The 3D framework of PPy wrapped on the graphene hydrogel nanocomposites demonstrated an outstanding capacitance retention for more than 4000 cycles, as well as a high specific capacitance of 375 F g^−1^. Recent supercapacitors with freestanding CNT/graphene/PPy hybrid electrodes showed a specific capacitance of 453 F g^−1^ and extremely high energy and power densities of 63 Wh kg^−1^ and 567 W kg^−1^, respectively, at a scan rate of 5 mV s^−1^ [86]. Nanowires, nanotubes and other hybrid graphene-PPy nanostructures, PPy/graphene film have been used [87]. Using 3D cellulose/PPy composites [88], TiO_2_/graphene/PPy composites [89], electrodes for supercapacitors have also been identified as PPy/SG composites [89] and PPy/rGO-CTAB composites [90]. As potential pseudocapacitor materials, graphene-PTh nanocomposites [91,92] and their derivatives have also been reported [92,93] At a current density of 0.1 A g^−1^, graphene-PEDOT and graphene-PHTh nanocomposites demonstrated specific capacitances of 800–1100 F g^−1^ [90]. Even after 2000 cycles, the specific capacitance of composite films made of PEDOT and rGO retains up to 90% of its initial value and exhibits high specific capacitance retention [94] Though attractive, conductive polymers have lower intrinsic conductivities than metals, which can result in slow electron/ion transport characteristics and poor rates of charge/discharge. In graphene/conductive polymer nanocomposites, the orientation of polymer chains can lead to increased electron transport and boost charge/discharge rates in supercapacitors. The ability to construct a complicated path for ions in the graphene composite structure that can result in irreversible specific capacitance losses is another significant benefit of using graphene/polymer nanocomposites in supercapacitors. Table 1 and Table 2 list the Graphene polymer nanocomposites that have been applied in batteries and supercapacitors. The predominant graphene polymer nanocomposites that have contributed significantly towards supercapacitor applications include: Graphene aerogel (GA) functionalized with *p*-phenylenediamine/PANI, rGO/PANI, Graphene (Gr)/poly (styrenesulfonic acid-graft polyaniline) (S-g-A), rGO/PANI fiber films, 3D multi-growth site graphene (MSG)/PANI, rGO/poly (3,4-ethylenedioxythiophene) (PEDOT), Polymer-wrapped rGO/nickel cobalt ferrite, Polymer-wrapped rGO/nickel cobalt ferrite and self-doped PANI/bonded graphene. Most of these systems employ rGO combined with PANI polymers, indicating this combination to be a better nanocomposite system for energy storage applications. 

## 4. Fullerene-Based Polymer Nanocomposites for Energy Storage

Table 3 shows the comparative physical properties of graphene versus fullerenes. Supercapacitors have been touted as effective energy storage options for cutting-edge electronic gadgets [127,128]. Supercapacitors are among the energy storage technologies that have high specific capacitance, power density and charged-discharge performance [129]. The three primary types of supercapacitors are double-layer capacitors with electrostatic charge storage, pseudocapacitors with electrochemical charge storage and hybrid capacitors with both electrochemical and electrostatic charge storage. Supercapacitors are among the energy storage technologies with the widest range of energy and power densities [130]. Supercapacitors with a higher energy density than batteries will be preferred in the future. The electron conduction characteristics of the polymer/fullerene nanomaterials are directly correlated with their specific capacitance. As a result, increasing the electrical conductivity of the nanocomposites is very important. The properties of electron conductivity and capacitance may be reduced by fullerene aggregation. The electrical conductivity was, thus, significantly improved using the fullerene/polymer nanocomposite systems, leading to fruitful interactions between polymer and fullerene nanofiller, providing upgraded attributes for high performance supercapacitors. The application of phase transition materials as energy storage materials is a promising direction [123]. High performance fullerene-based energy storage devices may be produced, particularly when environmentally friendly or bio-based components are used [131,132].

Between capacitors, batteries and fuel cells, supercapacitors have been proven to perform best [133,134]. The present demand for energy storage devices is seen in the modern electronics and automotive industries [46,135,136]. Electrochemical supercapacitors or pseudocapacitors have been considered as capable energy storage technologies [137,138], having low cost and large power storage [139,140]. When compared to conventional storage devices, these devices have been noted for their high resilience, high energy density and high power density [141,142,143], and are now being used with success in robotics, electronics and other fields [144,145,146]. 

Conjugated polymers like polyaniline, polypyrrole, polythiophene and poly(3,4-ethylenedioxythiophene) (PEDOT), have found usage in supercapacitors [146,147,148]. These conjugated polymers have excellent specific capacitance and electron conduction [149,150]. The low charge discharge, stability and reversibility of conducting polymers, however, may be a downside of their use in supercapacitors. As a result, nanoparticles including graphene, graphite and inorganic nanoparticles have been added to the conjugated polymers [151,152,153]. Due to their naturally high surface area and electrical conductivity, fullerenes have been investigated as potential materials for supercapacitor devices [154,155]. Supercapacitor designs have made use of fullerene polymeric nanocomposites [135,156]. Polyaniline emeraldine base (PANIEB) and polyaniline emeraldine base/fullerene C60 whisker (PANI-EB/FW) nanocomposites were created by Wang et al. [154]. The electrode for the supercapacitor was made of PANI-EB/FW. The specific capacitance of PANI-EB/FW was found to be significantly greater than the native PANI-EB (248 F g^−1^), and the nanocomposite electrode’s capacitance retention was 85.2% after 1500 cycles. The synergistic interactions between the polymer and fullerene nanoparticles were responsible for the remarkable performance. Additionally, PANI-EB and fullerene C60-based supercapacitor electrodes were created by Xiong et al. [157]. Para-phenylenediamine (PPD) and C60 were integrated to create a useful fullerene. Then, the in situ polymerization of aniline monomer incorporated the PPD functional C60, yielding a higher specific capacitance of 776 F g^−1^ after 50 cycles in comparison to the clean PANI-EB (492 F g^−1^). After 500 cycles, 96.5% and 94.9%, respectively, of the capacitance retention of the fullerene-based nanocomposite and nativePANI-EB were detected. The conjugated polymer’s conductivity and capacitance properties were improved by the C60’s electron retreating efficiency [158,159]. The PANI/PCBM nanocomposite was created by Ramadan et al. [160] using polyaniline (PANI) and phenyl-C60-butyric acid methyl ester (PCBM), the clean polyaniline’s specific capacitance was 1110 F g^−1^. With wt.% nanofiller, the capacitance was significantly increased to 2201 F g^−1^. Over 1000 cycles, 96% capacitance retention was noted. Due to the superior fusion of PANI and PCBM and the superior charge transport capabilities, such high values of specific capacitance were found. Supercapacitors have also benefited from poly(3-hexylthiophene) (P3HT) and PCBM-derived P3HT:PCBM nanocomposites [161,162]. Kausar (2022) has very recently published an elaborate review on fullerene applications in supercapacitors [163]. With respect to fullerenes, polyaniline emeraldine base/C60 nanocomposites were the most successful polymer nanocomposites for electrochemical energy storage applications (supercapacitor applications).

Conducting polymers with high electrical conductivity and capacitance, such as PANI, PTH, P3HT and PEDOT, can be used as supercapacitor electrodes [152,164]. The performance of the conjugated polymers in the supercapacitors was improved by blending with fullerene molecules. There is no doubt that fullerenes, when combined with polymer composites, could show enhanced properties. Although, the reports in this direction are not abundant, with the current inputs, we see that what lies ahead is definitely irresistible. Fullerenes- and Graphene-based polymer nanocomposites, compared to carbon nanomaterial-based polymers, are uniquely advanced, since both fullerenes and graphene are the most evolved forms of carbon nanomaterials. With their 2D, 3D structure, they do have a clear edge over the other carbon nanomaterials. More conclusive studies are needed to reveal the actual advantages when using fullerenes and graphene with respect to electrochemical energy storage applications. Since, as of now, there is insufficient information available to comment on this aspect. 

## 5. Future Recommendations and Conclusions

The current scenario of the milestones achieved in electrochemical energy storage harnessing the technology made available through carbon polymer nanocomposites, graphene polymer nanocomposites and fullerene polymer nanocomposites was reviewed. The up-to-date list of various graphene/fullerene polymer nanocomposites was listed. Most of the published work in this area revolved around polymers such as polyaniline (PANi), polypyrrole (PPy), poly(thiophene) (PTh), poly(hexylthiophene) (PHTh) and poly(3,4-ethylenedioxythiophene) (PEDOT), where PANi, PPy and PEDOT happened to predominate. We see that graphene/fullerene polymer nanocomposites were predominantly applied for supercapacitors, relatively less for batteries and even lesser for fuel cell applications. In all these applications, the inputs from the involvement of polymers show clear advantages. Yet, we note only scattered reports, supported by a handful of investigations. We recommend further exploration of this integrated field of graphene/fullerene/polymers. Moreover, the limitations of fullerenes, which include their susceptibility to breakdown in the presence of visible light and oxygen as well as their hydrophobic nature (leading to spontaneous agglomeration and limited solubility) have not been adequately addressed. These are definitive concerns and ought to be addressed. It is also known that most fullerene-based battery performance improvements are minimal; this should be looked into as well. Additionally, possible combinations with binary trinary composite systems to break these limitations faced by the standalone fullerene system should be tested. 

**Figure 4:**
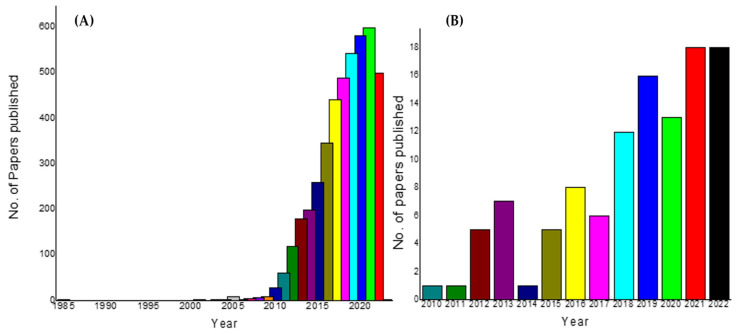
Results from a PubMed search using the terms (**A**) graphene and energy storage and (**B**) graphene polymer nanocomposites and energy storage.

Figure 4 represents the results from our PubMed search using the search terms ‘graphene and energy storage’ (Figure 4A), which made 4006 hits, while the search on ‘graphene polymer nanocomposites and energy storage’ yielded only 102 results (Figure 4B). This clearly indicates the low enthusiasm for graphene polymer nanocomposite applications for energy storage. We emphasize the need for increased application of the graphene polymer nanocomposites in the area of energy storage. To summarize, given that the inclusion of polymers into graphene and fullerenes has proven beneficial, what needs to be done is to promote and work out various permutation combinations of these composites to break down the limitations and barriers in H_2_ storage. These binary, ternary systems can offer more than the stand-alone graphene system alone.

This review emphasizes the need for involving more such polymers, particularly natural polymers such as chitin, cellulose and the like, for energy storage applications. Natural polymers such as chitosan, cellulose and their associated nanocomposites have been widely reported for various applications [165,166,167,168,169,170,171], yet have been sparsely reported for their ability to be used in energy storage. This is one area worth focusing on, given the fact they are naturally available, environmentally friendly, biocompatible and highly economical alternatives. Recent findings confirmed that cellulose, which is a highly abundant, versatile, sustainable and inexpensive material, can be used in the preparation of very stable and flexible electrochemical energy storage devices with high energy and power densities by using electrodes with high mass loadings, composed of conducting composites with high surface areas and thin layers of electroactive material, as well as cellulose-based current collectors and functional separators. There is definitely more to delve and much to harness from these as well as other natural polymers. There will no doubt be much more to hitch when these natural polymers are integrated with graphene/fullerene components to give rise to nanocomposites. This aspect deserves extensive research focus, which as on date, this review finds lacking. In addition to this, there are options from many graphene associates such as graphite, graphene oxide, graphane and the like. Additionally, C60 fullerene has been solely addressed, while other fullerene variants such as C70, C80 and C180 have not been attempted. These are some gaps that have been identified during the course of the review, which need to be reinforced and bridged-in with authentic research contributions.

Through the course of the review, we also found that there were no comparative studies that compared the efficiency of various graphene/fullerene polymer nanocomposites. Such a comparison would help determine which polymer nanocomposite system was the most efficient for electrochemical energy storage. Until the graphene/fullerene polymer systems are compared on a common platform, the optimized nanopolymer system cannot be identified. This is a key direction for future studies. Much has been done in terms of independent investigations involving CNT, graphene and fullerene, yet these need to be tested and their efficiencies and deficiencies compared and analyzed to arrive at conclusive statements. 

Moreover, to date, not many of these published carbon-based polymer nanocomposites have been practically implemented. There is a gap in the transfer of this acquired research knowledge to real-time applications. The fact that compared to polymer nanocomposites, carbon polymer nanocomposites were relatively less reported, and graphene/fullerene polymer nanocomposites even lesser reported, was evident during the review process. More studies, investigations and combinations need to be conducted in order to reach authoritative conclusions. These staggering ends in this area of research were identified during the review and highlighted here to draw the attention of the research community to bridge these gaps and make the most of this technology. Another relatively interesting area, which is yet to be extensively researched, is the area of biopolymers-based nanocomposites. Ramkumar and Minakshi’s research group has come up with a number of biopolymers for supercapacitor applications [172,173,174,175]. Combining biopolymer nanocomposites with graphene/fullerene nanomaterial is recommended as a future direction, which could prove beneficial. 

One of the major issues with polymer-based electrodes is that they are prone to structural degradation due to swelling and shrinking of conducting polymers during long term cycling, resulting in fading of electrochemical performance. A few reports suggest that this limitation can be overcome by nano structurization of conducting polymers [48] or blending with carbon-based materials, as carbon polymer nanocomposites [49,50,51] can significantly enhance the cycling stability. Although this has been explored, more conclusive studies are needed to work out the exact role of carbon nanomaterial in breaking this major limitation of conducting polymers. This will be a highly resourceful addition to the existing knowledge. Fundamental mechanistic studies are recommended in the future.

This review surveyed the status quo of graphene/fullerene nanocomposites for electrochemical energy storage options. The review identified that rather than the native graphene/fullerene nanomaterial, the polymer nanocomposite-integrated graphene/fullerenes were more suitable for hydrogen storage purposes. Polymer nanocomposites involving a combination of two or more entities invariably hold an added advantage of being able to overcome the limitations of the individual entities [176]. The potentials and possibilities from these nanocomposites were highlighted and the need to expand has been emphasized.

## Figures and Tables

**Table 1:** Graphene polymer nanocomposites for batteries.

Energy Storage Device	Carbon Materials	Polymer Composite	Electrochemical Performance	References
Lithium ion Batteries	Graphene sheets	poly(anthraquinonyl sulfide) and polyimide,	Ultrafast-Charge and -Discharge Cathodes	[60]
Li-ion/Na-ion batteries	Reduced Graphene oxide	radical polymer poly(2,2,6,6-tetramethylpiperidin-1-oxyl-4-yl methacrylate) (PTMA)	cathodes for Li-ion/Na-ion batteries with high energy storage	[95]
Na-ion batteries	Graphene sheets	polyimide nanorod composites(PInd)	superior capacity of 101.3 mAh g^–1^ at 500 mA g^−1^ after 1000 cycles.	[96]
Zinc-Ion Batteries	Reduced Graphene oxide	Polyethyleneimine + Quinone crystal	superior cyclability of charge/discharge cycle	[97]
Li-ion batteries	Reduced Graphene oxide	mesoporous polydopamine (mPDA),	superior cyclability of charge/discharge cycles	[98]
Li-ion/Na-ion batteries	Three-dimensional (3D) graphene framework	poly(anthraquinonyl sufide) (PAQS)	highest cathode charging and discharging capacity.	[99]
lithium-ion battery	Graphene oxide	polymer electrolytes (1) branched graft copolymer (BCP), poly(ethylene glycol) methyl ether methacrylate (PEGMA) (2) 3-(3,5,7,9,11,13,15-heptaisobutylpentacyclo-[9.5.1.13,9.15,15.17,13]octasiloxane-1-yl)propyl methacrylate (MA-POSS) (3) poly(ethylene glycol)-grafted graphene oxide (PGO)	Batteries with improved thermal and mechanical stabilities with PGO graphene nano hybrid.	[100]

**Table 2:** Supercapacitor milestones of graphene polymer nanocomposites.

Nanocomposites	Specific Capacitance (F g^−1^)	Current Density (A g^−1^)	Cycling Performances		Reference
			Capacity Retention (%)	Cycle Number	
Graphene oxide (GO)/pristine graphene (PG)/polyaniline (PANI)	793.7	1.0	83.8	1000	[101]
Graphene (Gr)/poly (styrenesulfonic acid-graft polyaniline) (S-g-A)	767	0.5	92	5000	[102]
Graphene aerogel (GA) functionalized with *p*-phenylenediamine/PANI	810	1.0	83.2	10,000	[103]
rGO/PANI film	763	0.34	76.5	2000	[104]
Flower-like PANI/graphene	1510	1.0	89	1500	[105]
Reduced graphene oxide (rGO)/PANI	952	1.0	88	1000	[106]
rGO/PANI	850	1.0	93.2	10,000	[107]
PANI/GO-vanadium (V)-ammonium persulfate (APS)	712	0.5	83	6000	[108]
rGO/PANI fiber films	1180	1.0	99	10,000	[109]
Graphene/PANI	311.3	0.4	88.6	1000	[110]
Graphene/PANI hydrogels	865.6	1.0	82	1000	[111]
3D multi-growth site graphene (MSG)/PANI	912	1.0	89.5	10,000	[112]
N-doped graphene/PANI hydrogels	514.3	1.0	84.7	1000	[113]
Unidirectional graphene aerogel (UGA)/PANI	538	1.0	74	1000	[114]
rGO/poly (3,4-ethylenedioxythiophene) (PEDOT)	202.7	1.0	90	9000	[114]
Holey nitrogen-doped graphene oxide (H-NGO)/PANI-10	746	1.0	97	2000	[115]
PANI/rGO-HT	420	0.2	80	6000	[116]
PANI/GO/copper (Cu)	557.9	1.0	88	1000	[117]
N, S-co-doped graphene hydrogel (N/SGH)/PANI	236.5	0.5	95.1	1000	[118]
Nitrogen-doped graphene/PANI-35%	620	0.5	87.4	5000	[119]
Graphene/PANI copolymer	1701.1	0.34	94.9	5000	[120]
3D porous graphene/PANI	542	1.14	82	3000	[121]
Polymer-wrapped rGO/nickel cobalt ferrite	1286	0.5	95	6000	[122]
Self-doped PANI/bonded graphene	642.6	1.0	100	5000	[123]
PANI/ /reduced crumbled GO	299	0.5	88.5	2000	[124]
GO/polypyrrole (PPy)/PANI	131	8.0	91	2000	[125]
Functionalized rGO/PANI	421	0.6	84.6	800	[126]

**Table 3:** Comparative listing of physical properties of Graphene and Fullerenes.

Physical Properties	Graphene	Fullerenes
Charge carrier mobility	~200,000 cm^2^/V·s	~1.2 × 10^−5^ cm^2^/V·s
Thermal conductivity	~5000 W/m·K	0.4 W/m·K
Transparency	~97.4%	~85%
Specific surface area	~2630 m^2^/g	~300 m^2^/g
Young’s modulus	~1 TPa	14 × 10^9^ Pa
Tensile strength	~1100 GPa	<100 GPa
Band gap	Zero	~1.4–3.5

## References

[1] Energy U.U.S. (2018). Energy Information Administration, Renewable Energy.

[2] Zhao P. (2019). Research on Power Transmission Direction and Scale of Interconnected Grid Considering Uncertainty. Master’s Thesis.

[3] Board E. (2020). The Growth of Electricity Sector in India from 1947–2020.

[4] Gielen D., Boshell F., Saygin D., Bazilian M.D., Wagner N., Gorini R. (2019). The Role of Renewable Energy in the Global Energy Transformation. Energy Strateg. Rev..

[5] Nelson J.P., Bolin W.D. (1995). Basics and Advances in Battery Systems. IEEE Trans. Ind. Appl..

[6] Anthony L.S., Vasudevan M., Perumal V., Ovinis M., Raja P.B., Edison T.N.J.I. (2021). Bioresource-Derived Polymer Composites for Energy Storage Applications: Brief Review. J. Environ. Chem. Eng..

[7] Kim T., Song W., Son D.-Y., Ono L.K., Qi Y. (2019). Lithium-Ion Batteries: Outlook on Present, Future, and Hybridized Technologies. J. Mater. Chem. A.

[8] Abdin Z., Khalilpour K.R. (2019). Single and Polystorage Technologies for Renewable-Based Hybrid Energy Systems. Polygeneration with Polystorage for Chemical and Energy Hubs.

[9] Zhao Y., Pohl O., Bhatt A.I., Collis G.E., Mahon P.J., Rüther T., Hollenkamp A.F. (2021). A Review on Battery Market Trends, Second-Life Reuse, and Recycling. Sustain. Chem..

[10] Evanko B., Boettcher S.W., Yoo S.J., Stucky G.D. (2017). Redox-Enhanced Electrochemical Capacitors: Status, Opportunity, and Best Practices for Performance Evaluation. ACS Energy Lett..

[11] Wang Y., Shi Z., Huang Y., Ma Y., Wang C., Chen M., Chen Y. (2009). Supercapacitor Devices Based on Graphene Materials. J. Phys. Chem. C.

[12] Velasco A., Ryu Y.K., Boscá A., Ladrón-de-Guevara A., Hunt E., Zuo J., Pedrós J., Calle F., Martinez J. (2021). Recent Trends in Graphene Supercapacitors: From Large Area to Microsupercapacitors. Sustain. Energy Fuels.

[13] Zhu J., Childress A.S., Karakaya M., Dandeliya S., Srivastava A., Lin Y., Rao A.M., Podila R. (2016). Defect-Engineered Graphene for High-Energy- and High-Power-Density Supercapacitor Devices. Adv. Mater..

[14] Zhang Y., Xie E. (2021). Functionalized and Tip-Open Carbon Nanotubes for High-Performance Symmetric Supercapacitors. Dalt. Trans..

[15] Lu Z., Raad R., Safaei F., Xi J., Liu Z., Foroughi J. (2019). Carbon Nanotube Based Fiber Supercapacitor as Wearable Energy Storage. Front. Mater..

[16] Komatsubara K., Suzuki H., Inoue H., Kishibuchi M., Takahashi S., Marui T., Umezawa S., Nakagawa T., Nasu K., Maetani M. (2022). Highly Oriented Carbon Nanotube Supercapacitors. ACS Appl. Nano Mater..

[17] Miao Y.-E., Huang Y., Zhang L., Fan W., Lai F., Liu T. (2015). Electrospun Porous Carbon Nanofiber@MoS 2 Core/Sheath Fiber Membranes as Highly Flexible and Binder-Free Anodes for Lithium-Ion Batteries. Nanoscale.

[18] Ding Q., Liu M., Miao Y.-E., Huang Y., Liu T. (2015). Electrospun Nickel-Decorated Carbon Nanofiber Membranes as Efficient Electrocatalysts for Hydrogen Evolution Reaction. Electrochim. Acta.

[19] Stoller M.D., Park S., Zhu Y., An J., Ruoff R.S. (2008). Graphene-Based Ultracapacitors. Nano Lett..

[20] Zhu Y., Murali S., Stoller M.D., Velamakanni A., Piner R.D., Ruoff R.S. (2010). Microwave Assisted Exfoliation and Reduction of Graphite Oxide for Ultracapacitors. Carbon N. Y..

[21] Zhu Y., Stoller M.D., Cai W., Velamakanni A., Piner R.D., Chen D., Ruoff R.S. (2010). Exfoliation of Graphite Oxide in Propylene Carbonate and Thermal Reduction of the Resulting Graphene Oxide Platelets. ACS Nano.

[22] Beaudin M., Zareipour H., Schellenberglabe A., Rosehart W. (2010). Energy Storage for Mitigating the Variability of Renewable Electricity Sources: An Updated Review. Energy Sustain. Dev..

[23] Arani A.A.K., Karami H., Gharehpetian G.B., Hejazi M.S.A. (2017). Review of Flywheel Energy Storage Systems Structures and Applications in Power Systems and Microgrids. Renew. Sustain. Energy Rev..

[24] Elliman R., Gould C., Al-Tai M. (2015). Review of Current and Future Electrical Energy Storage Devices. Proceedings of the 2015 50th International Universities Power Engineering Conference (UPEC).

[25] Hannan M.A., Hoque M.M., Mohamed A., Ayob A. (2017). Review of Energy Storage Systems for Electric Vehicle Applications: Issues and Challenges. Renew. Sustain. Energy Rev..

[26] Abbasi H., Antunes M., Velasco J.I. (2019). Recent Advances in Carbon-Based Polymer Nanocomposites for Electromagnetic Interference Shielding. Prog. Mater. Sci..

[27] Mohan V.B., Lau K., Hui D., Bhattacharyya D. (2018). Graphene-Based Materials and Their Composites: A Review on Production, Applications and Product Limitations. Compos. Part B Eng..

[28] Dang Z.-M., Yuan J.-K., Yao S.-H., Liao R.-J. (2013). Flexible Nanodielectric Materials with High Permittivity for Power Energy Storage. Adv. Mater..

[29] Devi N., Ghosh S., Perla V.K., Pal T., Mallick K. (2019). Laboratory Based Synthesis of the Pure Form of Gananite (BiF 3 ) Nanoparticles: A Potential Material for Electrochemical Supercapacitor Application. New J. Chem..

[30] Al-Saleh M.H. (2015). Electrically Conductive Carbon Nanotube/Polypropylene Nanocomposite with Improved Mechanical Properties. Mater. Des..

[31] Tang H., Chen G.-X., Li Q. (2016). Epoxy-Based High-k Composites with Low Dielectric Loss Caused by Reactive Core-Shell-Structured Carbon Nanotube Hybrids. Mater. Lett..

[32] Baek J.E., Kim J.Y., Jin H.M., Kim B.H., Lee K.E., Kim S.O. (2017). Single-Step Self-Assembly of Multilayer Graphene Based Dielectric Nanostructures. FlatChem.

[33] Lin B., Li Z.-T., Yang Y., Li Y., Lin J.-C., Zheng X.-M., He F.-A., Lam K.-H. (2019). Enhanced Dielectric Permittivity in Surface-Modified Graphene/PVDF Composites Prepared by an Electrospinning-Hot Pressing Method. Compos. Sci. Technol..

[34] Mohanapriya M.K., Deshmukh K., Chidambaram K., Ahamed M.B., Sadasivuni K.K., Ponnamma D., AlMaadeed M.A.-A., Deshmukh R.R., Pasha S.K.K. (2017). Polyvinyl Alcohol (PVA)/Polystyrene Sulfonic Acid (PSSA)/Carbon Black Nanocomposite for Flexible Energy Storage Device Applications. J. Mater. Sci. Mater. Electron..

[35] Fan Z., Wang D., Yuan Y., Wang Y., Cheng Z., Liu Y., Xie Z. (2020). A Lightweight and Conductive MXene/Graphene Hybrid Foam for Superior Electromagnetic Interference Shielding. Chem. Eng. J..

[36] Al-Saleh M.H. (2019). Carbon-Based Polymer Nanocomposites as Dielectric Energy Storage Materials. Nanotechnology.

[37] Cai C., Liu L., Fu Y. (2019). Processable Conductive and Mechanically Reinforced Polylactide/Graphene Bionanocomposites through Interfacial Compatibilizer. Polym. Compos..

[38] Ishaq S., Moussa M., Kanwal F., Ehsan M., Saleem M., Van T.N., Losic D. (2019). Facile Synthesis of Ternary Graphene Nanocomposites with Doped Metal Oxide and Conductive Polymers as Electrode Materials for High Performance Supercapacitors. Sci. Rep..

[39] Idumah C.I., Hassan A. (2016). Emerging Trends in Graphene Carbon Based Polymer Nanocomposites and Applications. Rev. Chem. Eng..

[40] Miller E.E., Hua Y., Tezel F.H. (2018). Materials for Energy Storage: Review of Electrode Materials and Methods of Increasing Capacitance for Supercapacitors. J. Energy Storage.

[41] Muzaffar A., Ahamed M.B., Deshmukh K., Thirumalai J. (2019). A Review on Recent Advances in Hybrid Supercapacitors: Design, Fabrication and Applications. Renew. Sustain. Energy Rev..

[42] Farzana R., Rajarao R., Bhat B.R., Sahajwalla V. (2018). Performance of an Activated Carbon Supercapacitor Electrode Synthesised from Waste Compact Discs (CDs). J. Ind. Eng. Chem..

[43] Tian J., Wu S., Yin X., Wu W. (2019). Novel Preparation of Hydrophilic Graphene/Graphene Oxide Nanosheets for Supercapacitor Electrode. Appl. Surf. Sci..

[44] Zhou Y., Zhou X., Ge C., Zhou W., Zhu Y., Xu B. (2019). Branched Carbon Nanotube/Carbon Nanofiber Composite for Supercapacitor Electrodes. Mater. Lett..

[45] Genc R., Alas M.O., Harputlu E., Repp S., Kremer N., Castellano M., Colak S.G., Ocakoglu K., Erdem E. (2017). High-Capacitance Hybrid Supercapacitor Based on Multi-Colored Fluorescent Carbon-Dots. Sci. Rep..

[46] Winter M., Brodd R.J. (2004). What Are Batteries, Fuel Cells, and Supercapacitors?. Chem. Rev..

[47] Chen T.-W., Kalimuthu P., Veerakumar P., Lin K.-C., Chen S.-M., Ramachandran R., Mariyappan V., Chitra S. (2022). Recent Developments in Carbon-Based Nanocomposites for Fuel Cell Applications: A Review. Molecules.

[48] Siwal S.S., Zhang Q., Devi N., Thakur V.K. (2020). Carbon-Based Polymer Nanocomposite for High-Performance Energy Storage Applications. Polymers.

[49] Sarang K.T., Miranda A., An H., Oh E.-S., Verduzco R., Lutkenhaus J.L. (2019). Poly(Fluorene- Alt -Naphthalene Diimide) as n-Type Polymer Electrodes for Energy Storage. ACS Appl. Polym. Mater..

[50] Li J., Han S., Zhang C., Wei W., Gu M., Meng L. (2019). High-Performance and Reactivation Characteristics of High-Quality, Graphene-Supported SnS 2 Heterojunctions for a Lithium-Ion Battery Anode. ACS Appl. Mater. Interfaces.

[51] Han H., Lee S.W., Moon K.H., Cho S. (2019). Fabrication of Solid-State Asymmetric Supercapacitors Based on Aniline Oligomers and Graphene Electrodes with Enhanced Electrochemical Performances. ACS Omega.

[52] Han C., Shi R., Zhou D., Li H., Xu L., Zhang T., Li J., Kang F., Wang G., Li B. (2019). High-Energy and High-Power Nonaqueous Lithium-Ion Capacitors Based on Polypyrrole/Carbon Nanotube Composites as Pseudocapacitive Cathodes. ACS Appl. Mater. Interfaces.

[53] Li X., Zhao T., Chen Q., Li P., Wang K., Zhong M., Wei J., Wu D., Wei B., Zhu H. (2013). Flexible All Solid-State Supercapacitors Based on Chemical Vapor Deposition Derived Graphene Fibers. Phys. Chem. Chem. Phys..

[54] Chen J., Xu J., Zhou S., Zhao N., Wong C.-P. (2015). Facile and Scalable Fabrication of Three-Dimensional Cu(OH) 2 Nanoporous Nanorods for Solid-State Supercapacitors. J. Mater. Chem. A.

[55] Khomenko V., Frackowiak E., Béguin F. (2005). Determination of the Specific Capacitance of Conducting Polymer/Nanotubes Composite Electrodes Using Different Cell Configurations. Electrochim. Acta.

[56] Fan L.-Z., Hu Y.-S., Maier J., Adelhelm P., Smarsly B., Antonietti M. (2007). High Electroactivity of Polyaniline in Supercapacitors by Using a Hierarchically Porous Carbon Monolith as a Support. Adv. Funct. Mater..

[57] Fan H., Liu W., Shen W. (2017). Honeycomb-like Composite Structure for Advanced Solid State Asymmetric Supercapacitors. Chem. Eng. J..

[58] Guo W., Yin Y.-X., Xin S., Guo Y.-G., Wan L.-J. (2012). Superior Radical Polymer Cathode Material with a Two-Electron Process Redox Reaction Promoted by Graphene. Energy Environ. Sci..

[59] Sun M., Li H., Wang J., Wang G. (2015). Promising Graphene/Carbon Nanotube Foam@π-Conjugated Polymer Self-Supporting Composite Cathodes for High-Performance Rechargeable Lithium Batteries. Carbon N. Y..

[60] Song Z., Xu T., Gordin M.L., Jiang Y.-B., Bae I.-T., Xiao Q., Zhan H., Liu J., Wang D. (2012). Polymer–Graphene Nanocomposites as Ultrafast-Charge and -Discharge Cathodes for Rechargeable Lithium Batteries. Nano Lett..

[61] Ji L., Meduri P., Agubra V., Xiao X., Alcoutlabi M. (2016). Graphene-Based Nanocomposites for Energy Storage. Adv. Energy Mater..

[62] Ke Q., Wang J. (2016). Graphene-Based Materials for Supercapacitor Electrodes–A Review. J. Mater..

[63] Xiao F., Yang S., Zhang Z., Liu H., Xiao J., Wan L., Luo J., Wang S., Liu Y. (2015). Scalable Synthesis of Freestanding Sandwich-Structured Graphene/Polyaniline/Graphene Nanocomposite Paper for Flexible All-Solid-State Supercapacitor. Sci. Rep..

[64] Biswas S., Drzal L.T. (2010). Multilayered Nanoarchitecture of Graphene Nanosheets and Polypyrrole Nanowires for High Performance Supercapacitor Electrodes. Chem. Mater..

[65] Park J.H., Ko J.M., Park O.O., Kim D.-W. (2002). Capacitance Properties of Graphite/Polypyrrole Composite Electrode Prepared by Chemical Polymerization of Pyrrole on Graphite Fiber. J. Power Sources.

[66] Chee W.K., Lim H.N., Harrison I., Chong K.F., Zainal Z., Ng C.H., Huang N.M. (2015). Performance of Flexible and Binderless Polypyrrole/Graphene Oxide/Zinc Oxide Supercapacitor Electrode in a Symmetrical Two-Electrode Configuration. Electrochim. Acta.

[67] Lim Y.S., Tan Y.P., Lim H.N., Huang N.M., Tan W.T., Yarmo M.A., Yin C.-Y. (2014). Potentiostatically Deposited Polypyrrole/Graphene Decorated Nano-Manganese Oxide Ternary Film for Supercapacitors. Ceram. Int..

[68] Xiong P., Huang H., Wang X. (2014). Design and Synthesis of Ternary Cobalt Ferrite/Graphene/Polyaniline Hierarchical Nanocomposites for High-Performance Supercapacitors. J. Power Sources.

[69] Guo X., Feng B., Gai L., Zhou J. (2019). Reduced Graphene Oxide/Polymer Dots-Based Flexible Symmetric Supercapacitors Delivering an Output Potential of 1.7 V with Electrochemical Charge Injection. Electrochim. Acta.

[70] Akhina H., Mohammed Arif P., Gopinathan Nair M.R., Nandakumar K., Thomas S. (2019). Development of Plasticized Poly (Vinyl Chloride)/Reduced Graphene Oxide Nanocomposites for Energy Storage Applications. Polym. Test..

[71] Suneetha R.B., Selvi P., Vedhi C. (2019). Synthesis, Structural and Electrochemical Characterization of Zn Doped Iron Oxide/Grapheneoxide/Chitosan Nanocomposite for Supercapacitor Application. Vacuum.

[72] Chabi S., Peng C., Yang Z., Xia Y., Zhu Y. (2015). Three Dimensional (3D) Flexible Graphene Foam/Polypyrrole Composite: Towards Highly Efficient Supercapacitors. RSC Adv..

[73] Azizi E., Arjomandi J., Lee J.Y. (2019). Reduced Graphene Oxide/Poly(1,5 Dihydroxynaphthalene)/TiO_2_ Nanocomposite Conducting Polymer Coated on Gold as a Supercapacitor Electrode. Electrochim. Acta.

[74] Pan Q., Zhao J., Qu W., Liu R., Li N., Xing B., Jiang S., Pang M., Zhao L., Zhang Y. (2019). Facile Synthesis of the 3D Framework Si@N-Doped C/Reduced Graphene Oxide Composite by Polymer Network Method for Highly Stable Lithium Storage. J. Phys. Chem. Solids.

[75] Arukula R., Vinothkannan M., Kim A.R., Yoo D.J. (2019). Cumulative Effect of Bimetallic Alloy, Conductive Polymer and Graphene toward Electrooxidation of Methanol: An Efficient Anode Catalyst for Direct Methanol Fuel Cells. J. Alloys Compd..

[76] Baruah B., Kumar A. (2018). PEDOT:PSS/MnO_2_/RGO Ternary Nanocomposite Based Anode Catalyst for Enhanced Electrocatalytic Activity of Methanol Oxidation for Direct Methanol Fuel Cell. Synth. Met..

[77] Wang D.-W., Li F., Zhao J., Ren W., Chen Z.-G., Tan J., Wu Z.-S., Gentle I., Lu G.Q., Cheng H.-M. (2009). Fabrication of Graphene/Polyaniline Composite Paper via In Situ Anodic Electropolymerization for High-Performance Flexible Electrode. ACS Nano.

[78] Gómez H., Ram M.K., Alvi F., Villalba P., Stefanakos E.L., Kumar A. (2011). Graphene-Conducting Polymer Nanocomposite as Novel Electrode for Supercapacitors. J. Power Sources.

[79] Liu X., Zheng Y., Wang X. (2015). Controllable Preparation of Polyaniline-Graphene Nanocomposites Using Functionalized Graphene for Supercapacitor Electrodes. Chemistry.

[80] Yan J., Wei T., Shao B., Fan Z., Qian W., Zhang M., Wei F. (2010). Preparation of a Graphene Nanosheet/Polyaniline Composite with High Specific Capacitance. Carbon N. Y..

[81] Xu J., Wang K., Zu S.-Z., Han B.-H., Wei Z. (2010). Hierarchical Nanocomposites of Polyaniline Nanowire Arrays on Graphene Oxide Sheets with Synergistic Effect for Energy Storage. ACS Nano.

[82] Liu X., Wen N., Wang X., Zheng Y. (2015). A High-Performance Hierarchical Graphene@Polyaniline@Graphene Sandwich Containing Hollow Structures for Supercapacitor Electrodes. ACS Sustain. Chem. Eng..

[83] Chi K., Zhang Z., Xi J., Huang Y., Xiao F., Wang S., Liu Y. (2014). Freestanding Graphene Paper Supported Three-Dimensional Porous Graphene–Polyaniline Nanocomposite Synthesized by Inkjet Printing and in Flexible All-Solid-State Supercapacitor. ACS Appl. Mater. Interfaces.

[84] Xu D., Xu Q., Wang K., Chen J., Chen Z. (2014). Fabrication of Free-Standing Hierarchical Carbon Nanofiber/Graphene Oxide/Polyaniline Films for Supercapacitors. ACS Appl. Mater. Interfaces.

[85] Liu A., Li C., Bai H., Shi G. (2010). Electrochemical Deposition of Polypyrrole/Sulfonated Graphene Composite Films. J. Phys. Chem. C.

[86] Aphale A., Maisuria K., Mahapatra M.K., Santiago A., Singh P., Patra P. (2015). Hybrid Electrodes by In-Situ Integration of Graphene and Carbon-Nanotubes in Polypyrrole for Supercapacitors. Sci. Rep..

[87] de Oliveira H.P., Sydlik S.A., Swager T.M. (2013). Supercapacitors from Free-Standing Polypyrrole/Graphene Nanocomposites. J. Phys. Chem. C.

[88] Liu Y., Zhou J., Tang J., Tang W. (2015). Three-Dimensional, Chemically Bonded Polypyrrole/Bacterial Cellulose/Graphene Composites for High-Performance Supercapacitors. Chem. Mater..

[89] Jiang L., Lu X., Xie C., Wan G., Zhang H., Youhong T. (2015). Flexible, Free-Standing TiO 2 –Graphene–Polypyrrole Composite Films as Electrodes for Supercapacitors. J. Phys. Chem. C.

[90] Fan X., Yang Z., He N. (2015). Hierarchical Nanostructured Polypyrrole/Graphene Composites as Supercapacitor Electrode. RSC Adv..

[91] Alvi F., Basnayaka P.A., Ram M.K., Gomez H., Stefanako E., Goswami Y., Kumar A. (2011). Graphene-Polythiophene Nanocomposite as Novel Supercapacitor Electrode Material. J. New Mater. Electrochem. Syst..

[92] Alvi F., Ram M.K., Basnayaka P., Stefanakos E., Goswami Y., Hoff A., Kumar A. (2011). Electrochemical Supercapacitors Based on Graphene-Conducting Polythiophenes Nanocomposite. ECS Trans..

[93] Alvi F., Ram M.K., Basnayaka P.A., Stefanakos E., Goswami Y., Kumar A. (2011). Graphene–Polyethylenedioxythiophene Conducting Polymer Nanocomposite Based Supercapacitor. Electrochim. Acta.

[94] Lehtimäki S., Suominen M., Damlin P., Tuukkanen S., Kvarnström C., Lupo D. (2015). Preparation of Supercapacitors on Flexible Substrates with Electrodeposited PEDOT/Graphene Composites. ACS Appl. Mater. Interfaces.

[95] Jin W., Zhou T., Wang Z., Xue W., Feng C., Zhang F., Huang X., Yang D., Théato P., Li Y. (2021). Radical Polymer Grafted Graphene for High-Performance Li+/Na+ Organic Cathodes. J. Power Sources.

[96] Ma J., Kong Y., Luo Y., Huang Y., Han S. (2021). Flexible Polyimide Nanorod/Graphene Framework as an Organic Cathode for Rechargeable Sodium-Ion Batteries. J. Phys. Chem. C.

[97] Kobayashi H., Oizumi K., Tomai T., Honma I. (2022). Graphene and Polyethyleneimine Bilayer Wrapping onto Quinone Molecular Crystal Cathode Materials for Aqueous Zinc-Ion Batteries. ACS Appl. Energy Mater..

[98] Wang N., Hou D., Li Q., Zhang P., Wei H., Mai Y. (2019). Two-Dimensional Interface Engineering of Mesoporous Polydopamine on Graphene for Novel Organic Cathodes. ACS Appl. Energy Mater..

[99] Zhang Y., Huang Y., Yang G., Bu F., Li K., Shakir I., Xu Y. (2017). Dispersion–Assembly Approach to Synthesize Three-Dimensional Graphene/Polymer Composite Aerogel as a Powerful Organic Cathode for Rechargeable Li and Na Batteries. ACS Appl. Mater. Interfaces.

[100] Shim J., Kim D.-G., Kim H.J., Lee J.H., Baik J.-H., Lee J.-C. (2014). Novel Composite Polymer Electrolytes Containing Poly(Ethylene Glycol)-Grafted Graphene Oxide for All-Solid-State Lithium-Ion Battery Applications. J. Mater. Chem. A.

[101] Zhang Y., Si L., Zhou B., Zhao B., Zhu Y., Zhu L., Jiang X. (2016). Synthesis of Novel Graphene Oxide/Pristine Graphene/Polyaniline Ternary Composites and Application to Supercapacitor. Chem. Eng. J..

[102] Lee J.W., Lee J.U., Jo J.W., Bae S., Kim K.T., Jo W.H. (2016). In-Situ Preparation of Graphene/Poly(Styrenesulfonic Acid-Graft-Polyaniline) Nanocomposite via Direct Exfoliation of Graphite for Supercapacitor Application. Carbon N. Y..

[103] Bulin C., Yu H., Ge X., Xin G., Xing R., Li R., Zhang B. (2017). Preparation and Supercapacitor Performance of Functionalized Graphene Aerogel Loaded with Polyaniline as a Freestanding Electrode. J. Mater. Sci..

[104] Hong X., Zhang B., Murphy E., Zou J., Kim F. (2017). Three-Dimensional Reduced Graphene Oxide/Polyaniline Nanocomposite Film Prepared by Diffusion Driven Layer-by-Layer Assembly for High-Performance Supercapacitors. J. Power Sources.

[105] Ke F., Liu Y., Xu H., Ma Y., Guang S., Zhang F., Lin N., Ye M., Lin Y., Liu X. (2017). Flower-like Polyaniline/Graphene Hybrids for High-Performance Supercapacitor. Compos. Sci. Technol..

[106] Li R. (2017). Graphene-Enabled Improved Supercapacitor Performance of Polyaniline Nanofiber Composites. Int. J. Electrochem. Sci..

[107] Qin G., Zhang H., Liao H., Li Z., Tian J., Lin Y., Zhang D., Wu Q. (2017). Novel Graphene Nanosheet-Wrapped Polyaniline Rectangular-like Nanotubes for Flexible All-Solid-State Supercapacitors. J. Mater. Sci..

[108] Tabrizi A.G., Arsalani N., Namazi H., Ahadzadeh I. (2017). Vanadium Oxide Assisted Synthesis of Polyaniline Nanoarrays on Graphene Oxide Sheets and Its Application in Supercapacitors. J. Electroanal. Chem..

[109] Zhang L., Huang D., Hu N., Yang C., Li M., Wei H., Yang Z., Su Y., Zhang Y. (2017). Three-Dimensional Structures of Graphene/Polyaniline Hybrid Films Constructed by Steamed Water for High-Performance Supercapacitors. J. Power Sources.

[110] Ates M., El-Kady M., Kaner R.B. (2018). Three-Dimensional Design and Fabrication of Reduced Graphene Oxide/Polyaniline Composite Hydrogel Electrodes for High Performance Electrochemical Supercapacitors. Nanotechnology.

[111] Ji J., Li R., Li H., Shu Y., Li Y., Qiu S., He C., Yang Y. (2018). Phytic Acid Assisted Fabrication of Graphene/Polyaniline Composite Hydrogels for High-Capacitance Supercapacitors. Compos. Part B Eng..

[112] Zheng X., Yu H., Xing R., Ge X., Sun H., Li R., Zhang Q. (2018). Multi-Growth Site Graphene/Polyaniline Composites with Highly Enhanced Specific Capacitance and Rate Capability for Supercapacitor Application. Electrochim. Acta.

[113] Zou Y., Zhang Z., Zhong W., Yang W. (2018). Hydrothermal Direct Synthesis of Polyaniline, Graphene/Polyaniline and N-Doped Graphene/Polyaniline Hydrogels for High Performance Flexible Supercapacitors. J. Mater. Chem. A.

[114] Wu X., Tang L., Zheng S., Huang Y., Yang J., Liu Z., Yang W., Yang M. (2018). Hierarchical Unidirectional Graphene Aerogel/Polyaniline Composite for High Performance Supercapacitors. J. Power Sources.

[115] Liu J., Du P., Wang Q., Liu D., Liu P. (2019). Mild Synthesis of Holey N-Doped Reduced Graphene Oxide and Its Double-Edged Effects in Polyaniline Hybrids for Supercapacitor Application. Electrochim. Acta.

[116] Moyseowicz A., Gryglewicz G. (2019). Hydrothermal-Assisted Synthesis of a Porous Polyaniline/Reduced Graphene Oxide Composite as a High-Performance Electrode Material for Supercapacitors. Compos. Part B Eng..

[117] Ma Y., Zhao D., Chen Y., Huang J., Zhang Z., Zhang X., Zhang B. (2019). A Novel Core-Shell Polyaniline/Graphene Oxide/Copper Nanocomposite for High Performance and Low-Cost Supercapacitors. Chem. Pap..

[118] Du X., Shi X., Li Y., Cao K. (2021). Construction of N, S-co-doped Graphene/Polyaniline Composite as Free-standing Electrode Material. Int. J. Energy Res..

[119] Ge M., Hao H., Lv Q., Wu J., Li W. (2020). Hierarchical Nanocomposite That Coupled Nitrogen-Doped Graphene with Aligned PANI Cores Arrays for High-Performance Supercapacitor. Electrochim. Acta.

[120] Li T., Wang X., Liu P., Yang B., Diao S., Gao Y. (2020). Synthesis of Graphene/Polyaniline Copolymer for Solid-State Supercapacitor. J. Electroanal. Chem..

[121] Feng H., Zhang F., Chen N., Tan L., Liu C., Hu D., Zhao D. (2021). Enhanced Capacitive Performance of Polyaniline on Hydroquinone-Functionalized Three-Dimensional Porous Graphene Substrate for Supercapacitors. J. Mater. Sci. Mater. Electron..

[122] Hareesh K., Rondiya S.R., Dzade N.Y., Dhole S.D., Williams J., Sergey S. (2021). Polymer-Wrapped Reduced Graphene Oxide/Nickel Cobalt Ferrite Nanocomposites as Tertiary Hybrid Supercapacitors: Insights from Experiment and Simulation. J. Sci. Adv. Mater. Devices.

[123] Kung C.-Y., Wang T.-L., Lin H.-Y., Yang C.-H. (2021). A High-Performance Covalently Bonded Self-Doped Polyaniline–Graphene Assembly Film with Superior Stability for Supercapacitors. J. Power Sources.

[124] Macherla N., Singh K., Santosh M.S., Kumari K., Lekkala R.G.R. (2021). Heat Assisted Facile Synthesis of Nanostructured Polyaniline/Reduced Crumbled Graphene Oxide as a High-Performance Flexible Electrode Material for Supercapacitors. Colloids Surf. A Physicochem. Eng. Asp..

[125] Zhao Z., Wang H., Huang H., Li L., Yu X. (2021). Graphene Oxide/Polypyrrole/Polyaniline Composite Hydrogel Synthesized by Vapor-Liquid Interfacial Method for Supercapacitors. Colloids Surfaces A Physicochem. Eng. Asp..

[126] Wang Y., Wang Y., Xu X., Wang C. (2021). Facile Route for the Preparation of Functionalized Reduced Graphene Oxide/Polyaniline Composite and Its Enhanced Electrochemical Performance. ECS J. Solid State Sci. Technol..

[127] Naoi K., Naoi W., Aoyagi S., Miyamoto J., Kamino T. (2013). New Generation “Nanohybrid Supercapacitor Technology”. Acc. Chem. Res..

[128] Raza W., Ali F., Raza N., Luo Y., Kim K.-H., Yang J., Kumar S., Mehmood A., Kwon E.E. (2018). Recent Advancements in Supercapacitor Technology. Nano Energy.

[129] Simon P., Gogotsi Y. (2008). Materials for electrochemical capacitors. Nat Mater..

[130] Libich J., Máca J., Vondrák J., Čech O., Sedlaříková M. (2018). Supercapacitors: Properties and Applications. J. Energy Storage.

[131] Gong S., Li X., Sheng M., Liu S., Zheng Y., Wu H., Lu X., Qu J. (2021). High Thermal Conductivity and Mechanical Strength Phase Change Composite with Double Supporting Skeletons for Industrial Waste Heat Recovery. ACS Appl. Mater. Interfaces.

[132] Yin G.-Z., Yang X.-M., Hobson J., López A.M., Wang D.-Y. (2022). Bio-Based Poly (Glycerol-Itaconic Acid)/PEG/APP as Form Stable and Flame-Retardant Phase Change Materials. Compos. Commun..

[133] Conway B.E. (1999). Similarities and Differences between Supercapacitors and Batteries for Storing Electrical Energy. Electrochemical Supercapacitors.

[134] Hu C.-C., Chang K.-H., Lin M.-C., Wu Y.-T. (2006). Design and Tailoring of the Nanotubular Arrayed Architecture of Hydrous RuO 2 for Next Generation Supercapacitors. Nano Lett..

[135] Pech D., Brunet M., Durou H., Huang P., Mochalin V., Gogotsi Y., Taberna P.-L., Simon P. (2010). Ultrahigh-Power Micrometre-Sized Supercapacitors Based on Onion-like Carbon. Nat. Nanotechnol..

[136] Larcher D., Tarascon J.-M. (2015). Towards Greener and More Sustainable Batteries for Electrical Energy Storage. Nat. Chem..

[137] Mousty C., Leroux F. (2012). LDHs as Electrode Materials for Electrochemical Detection and Energy Storage: Supercapacitor, Battery and (Bio)-Sensor. Recent Pat. Nanotechnol..

[138] Chen G.Z. (2017). Supercapacitor and Supercapattery as Emerging Electrochemical Energy Stores. Int. Mater. Rev..

[139] Bao L., Zang J., Li X. (2011). Flexible Zn_2_SnO_4_/MnO_2_ Core/Shell Nanocable−Carbon Microfiber Hybrid Composites for High-Performance Supercapacitor Electrodes. Nano Lett..

[140] Chen Z., Augustyn V., Wen J., Zhang Y., Shen M., Dunn B., Lu Y. (2011). High-Performance Supercapacitors Based on Intertwined CNT/V_2_O^5^ Nanowire Nanocomposites. Adv. Mater..

[141] Ryu K.S., Lee Y.-G., Hong Y.-S., Park Y.J., Wu X., Kim K.M., Kang M.G., Park N.-G., Chang S.H. (2004). Poly(Ethylenedioxythiophene) (PEDOT) as Polymer Electrode in Redox Supercapacitor. Electrochim. Acta.

[142] Kim Y.-T., Tadai K., Mitani T. (2005). Highly Dispersed Ruthenium Oxide Nanoparticles on Carboxylated Carbon Nanotubes for Supercapacitor Electrode Materials. J. Mater. Chem..

[143] Sen P., De A., Chowdhury A.D., Bandyopadhyay S.K., Agnihotri N., Mukherjee M. (2013). Conducting Polymer Based Manganese Dioxide Nanocomposite as Supercapacitor. Electrochim. Acta.

[144] Wang J., Xu Y., Chen X., Du X. (2007). Electrochemical Supercapacitor Electrode Material Based on Poly(3,4-Ethylenedioxythiophene)/Polypyrrole Composite. J. Power Sources.

[145] Chen H.-W., Hsu C.-Y., Chen J.-G., Lee K.-M., Wang C.-C., Huang K.-C., Ho K.-C. (2010). Plastic Dye-Sensitized Photo-Supercapacitor Using Electrophoretic Deposition and Compression Methods. J. Power Sources.

[146] White A.M., Slade R.C.T. (2004). Electrochemically and Vapour Grown Electrode Coatings of Poly(3,4-Ethylenedioxythiophene) Doped with Heteropolyacids. Electrochim. Acta.

[147] Snook G.A., Peng C., Fray D.J., Chen G.Z. (2007). Achieving High Electrode Specific Capacitance with Materials of Low Mass Specific Capacitance: Potentiostatically Grown Thick Micro-Nanoporous PEDOT Films. Electrochem. Commun..

[148] Liu K., Hu Z., Xue R., Zhang J., Zhu J. (2008). Electropolymerization of High Stable Poly(3,4-Ethylenedioxythiophene) in Ionic Liquids and Its Potential Applications in Electrochemical Capacitor. J. Power Sources.

[149] Patake V.D., Lokhande C.D., Joo O.S. (2009). Electrodeposited Ruthenium Oxide Thin Films for Supercapacitor: Effect of Surface Treatments. Appl. Surf. Sci..

[150] Poizot P., Dolhem F. (2011). Clean Energy New Deal for a Sustainable World: From Non-CO_2_ Generating Energy Sources to Greener Electrochemical Storage Devices. Energy Environ. Sci..

[151] Stenger-Smith J.D., Webber C.K., Anderson N., Chafin A.P., Zong K., Reynolds J.R. (2002). Poly(3,4-Alkylenedioxythiophene)-Based Supercapacitors Using Ionic Liquids as Supporting Electrolytes. J. Electrochem. Soc..

[152] Jayalakshmi M., Rao M.M., Venugopal N., Kim K.-B. (2007). Hydrothermal Synthesis of SnO_2_–V_2_O_5_ Mixed Oxide and Electrochemical Screening of Carbon Nano-Tubes (CNT), V_2_O_5_, V_2_O_5_–CNT, and SnO_2_–V_2_O_5_–CNT Electrodes for Supercapacitor Applications. J. Power Sources.

[153] Zhang K., Zhang L.L., Zhao X.S., Wu J. (2010). Graphene/Polyaniline Nanofiber Composites as Supercapacitor Electrodes. Chem. Mater..

[154] Wang H., Yan X., Piao G. (2017). A High-Performance Supercapacitor Based on Fullerene C 60 Whisker and Polyaniline Emeraldine Base Composite. Electrochim. Acta.

[155] Benzigar M.R., Joseph S., Saianand G., Gopalan A.-I., Sarkar S., Srinivasan S., Park D.-H., Kim S., Talapaneni S.N., Ramadass K. (2019). Highly Ordered Iron Oxide-Mesoporous Fullerene Nanocomposites for Oxygen Reduction Reaction and Supercapacitor Applications. Microporous Mesoporous Mater..

[156] Lin J., Zhang C., Yan Z., Zhu Y., Peng Z., Hauge R.H., Natelson D., Tour J.M. (2013). 3-Dimensional Graphene Carbon Nanotube Carpet-Based Microsupercapacitors with High Electrochemical Performance. Nano Lett..

[157] Xiong S., Yang F., Jiang H., Ma J., Lu X. (2012). Covalently Bonded Polyaniline/Fullerene Hybrids with Coral-like Morphology for High-Performance Supercapacitor. Electrochim. Acta.

[158] Xiong S., Phua S.L., Dunn B.S., Ma J., Lu X. (2010). Covalently Bonded Polyaniline−TiO_2_ Hybrids: A Facile Approach to Highly Stable Anodic Electrochromic Materials with Low Oxidation Potentials. Chem. Mater..

[159] Dou F., Silva C., Zhang X. (2013). The Role of Acceptor-Rich Domain in Optoelectronic Properties of Photovoltaic Diodes Based on Polymer Blends. Chem. Phys. Lett..

[160] Ramadan A., Anas M., Ebrahim S., Soliman M., Abou-Aly A.I. (2020). Polyaniline/Fullerene Derivative Nanocomposite for Highly Efficient Supercapacitor Electrode. Int. J. Hydrogen Energy.

[161] Wang Y.Z., Wang Q., Xie H.Y., Ho L.P., Tan D.M.F., Diao Y.Y., Chen W., Xie X.N. (2012). Fabrication of Highly Ordered P3HT:PCBM Nanostructures and Its Application as a Supercapacitive Electrode. Nanoscale.

[162] Momodu D., Bello A., Dangbegnon J., Barzeger F., Fabiane M., Manyala N. (2015). P3HT:PCBM/Nickel-Aluminum Layered Double Hydroxide-Graphene Foam Composites for Supercapacitor Electrodes. J. Solid State Electrochem..

[163] Kausar A. (2022). Fullerene Reinforced Polymeric Nanocomposites for Energy Storage—Status and Prognoses. Front. Mater..

[164] Wu N.-L. (2002). Nanocrystalline Oxide Supercapacitors. Mater. Chem. Phys..

[165] Muthu M., Gopal J., Chun S., Devadoss A.J.P., Hasan N., Sivanesan I. (2021). Crustacean Waste-Derived Chitosan: Antioxidant Properties and Future Perspective. Antioxidants.

[166] Sivanesan I., Gopal J., Muthu M., Shin J., Mari S., Oh J. (2021). Green Synthesized Chitosan/Chitosan Nanoforms/Nanocomposites for Drug Delivery Applications. Polymers.

[167] Oh J.W., Shin J., Chun S., Muthu M., Gopal J. (2021). Evaluating the Anticarcinogenic Activity of Surface Modified/Functionalized Nanochitosan: The Emerging Trends and Endeavors. Polymers.

[168] Sivanesan I., Muthu M., Gopal J., Hasan N., Kashif Ali S., Shin J., Oh J.W. (2021). Nanochitosan: Commemorating the Metamorphosis of an ExoSkeletal Waste to a Versatile Nutraceutical. Nanomaterials.

[169] Gopal J., Muthu M., Dhakshanamurthy T., Kim K.J., Hasan N., Kwon S.J., Chun S. (2019). Sustainable ecofriendly phytoextract mediated one pot green recovery of chitosan. Sci. Rep..

[170] Gopal J., Abdelhamid H.N., Hua P.Y., Wu H.F. (2013). Chitosan nanomagnets for effective extraction and sensitive mass spectrometric detection of pathogenic bacterial endotoxin from human urine. J. Mater. Chem. B.

[171] Sivanesan I., Gopal J., Muthu M., Shin J., Oh J.W. (2021). Reviewing Chitin/Chitosan Nanofibers and Associated Nanocomposites and Their Attained Medical Milestones. Polymers.

[172] Ramkumar R., Minakshi M. (2015). Fabrication of ultrathin CoMoO_4_ nanosheets modified with chitosan and their improved performance in energy storage device. Dalton Trans..

[173] Minakshi M., Meyrick D., Appadoo D. (2013). Maricite (NaMn_1/3_Ni^1/3^Co_1/3_PO_4_)/activated carbon: Hybrid capacitor. Energy Fuels.

[174] Minakshi M., Duraisamy S., Tirupathi P., Kandhasamy S., Munichandraiah N. (2014). Multi-component olivine for lithium-ion hybrid capacitor. Int. J. Electrochem. Sci..

[175] Ramkumarar R., Sundaram M.M. (2016). A biopolymer gel-decorated cobalt molybdate nanowafer: Effective graft polymer cross-linked with an organic acid for better energy storage. New J. Chem..

[176] Revanappa S.K., Soni I., Siddalinganahalli M., Jayaprakash G.K., Flores-Moreno R., Bananakere Nanjegowda C. (2022). A Fukui Analysis of an Arginine-Modified Carbon Surface for the Electrochemical Sensing of Dopamine. Materials.

